# Supplementary material for: Insomnia Telemedicine OSCE (TeleOSCE): A Simulated Standardized Patient Video-Visit Case for Clerkship Students
Source: MedEdPORTAL. 2019 Dec 27;15:10867. doi: 10.15766/mep_2374-8265.10867 (PMC7012306; doi:10.15766/mep_2374-8265.10867)
Supplement: Supplementary file 1 — A. Standardized Patient Case.docx B. Student Scenario.docx C. Room Setup.pdf D. Checklist.docx E. ICS8 Competency Form.docx [file mep-15-10867-s001.zip › _Educational_Summary_Report_10867.pdf]

MedEdPORTAL® The AAMC Journal of Teaching and Learning Resources

Original Publication Open Access

Insomnia Telemedicine OSCE (TeleOSCE): A Simulated Standardized Patient Video-Visit Case for Clerkship Students

Rebecca E. Cantone, MD*, Ryan Palmer, EdD, MFA, Lisa Grill Dodson, MD, Frances E. Biagioli, MD

*Corresponding author: cantone@ohsu.edu

Abstract

Introduction: Telemedicine is a growing practice with minimal training in US medical schools. Telemedicine OSCE (TeleOSCE) simulations allow students to practice this type of patient interaction in a standardized way. **Methods:** The Insomnia–Rural TeleOSCE was implemented as part of a required clinical clerkship for students in their second, third, or fourth year of medical school. This case addressed a patient with depression in a medically underserved area. Students performed it as a formative experience and received immediate feedback. They then completed a survey to evaluate the experience. **Results:** Students ($n = 287$) rated the quality of the experience 7.59 out of 10. Comments showed that 61 learners thought the TeleOSCE was a positive experience, 35 wanted more teaching about telemedicine, 28 improved their understanding of barriers to care, 25 expressed concern over minimal other training, 23 found the TeleOSCE important and challenging, 16 appreciated the differences between in-person and remote visits, and 15 wanted fewer distractions. Eight students worried about how they would be judged, five learned from the technical limitations, five requested more time, five were skeptical of the utility, and five saw telemedicine as triage. **Discussion:** The TeleOSCE allows learners to gain exposure to telemedicine in a safe simulated teaching environment and assesses learner competencies. The TeleOSCE also improves students' understanding of barriers to care and the utility of telemedicine. It logistically allows faculty to directly assess distance students on their clinical reasoning and patient communication skills.

Keywords
Telemedicine, Standardized Patient, OSCE, Rural, Insomnia, Clinical Skills Assessment, Computer-Based Simulation, Family Medicine, Psychiatry, Simulation

Educational Objectives

By the end of this simulation, learners will be able to:

1. Incorporate geographic limitations of care into patient care plans.
2. Demonstrate the provision of clinical care remotely via telemedicine.
3. Use clinical decision support tools to improve the care of patients.
4. Utilize telemedicine video technology to clinically assess a patient with insomnia.
5. Identify the care needed for a patient with depression.

Citation:
Cantone RE, Palmer R, Dodson LG, Biagioli FE. Insomnia telemedicine OSCE (TeleOSCE): a simulated standardized patient video-visit case for clerkship students. *MedEdPORTAL*. 2019;15:10867. https://doi.org/10.15766/mep_2374-8265.10867

Introduction

Telemedicine is an emerging model for health care delivery, and 90% of health care executives have reported developing or having implemented a telemedicine program.[1] Unfortunately, few medical schools offer this training to students (learners) prior to their entry into the workforce.[2] Simulation may be a way to create this exposure.

Simulation is a widely used model of medical training, allowing incorporation of practical, hands-on skills training outside of the classroom.[3] Shortridge et al.[4] discussed the need for rural telemedicine in Oklahoma, which echoes the need throughout the US.[5] We have implemented a telemedicine simulation curriculum in our family medicine core clinical experience (clerkship) as a mechanism to train future providers to practice in rural or remote locations where patients may not be able to reach a health care facility. Additionally, our curriculum accommodates learners at off-campus sites in the community, allowing remote observation of students' skill development by trained faculty in a standardized setting. It is

Copyright © 2019 Cantone et al. This is an open-access publication distributed under the terms of the Creative Commons Attribution license.

focused on providing exposure and feedback to learners in a safe environment that is free of formal grades reported in their clerkship. Our team found that participants in the telemedicine objective structured clinical examination (TeleOSCE) had statistically significant improvements in their knowledge and confidence in telemedicine versus students who did not have this experience.[6]

We found no similar telemedicine cases through a review of the literature and *MedEdPORTAL* curricula. Although there were trainings on how to utilize the technology, we could not find examples that incorporated medical students, telemedicine software, standardized patients (SPs), and feedback on using the software targeted at building rapport with patients. One of our own cases utilizing the same curricular goals but different medical decision making was published with the Society of Teachers of Family Medicine.[7] This is therefore an identified gap in medical education, particularly given the growing practice of telemedicine in clinical care. Even though students may not have real-world experiences with telemedicine during their curriculum, they are able to access urgent care as a patient at our institution via telemedicine technology. The telemedicine cases assess both the use of telemedicine technology and the ability to engage a patient in a telemedicine encounter; they are adaptable to both on- and off-campus learners.

Methods

We implemented the TeleOSCE as a formative tool, given in week 2 of a 4-week clerkship. Our clerkship is a required rotation that is taken during medical students' second, third, or fourth year in a competency-based medical education model. We aimed to address several of the AAMC's Core Entrustable Professional Activities for Entering Residency[8] and build telemedicine practice skills not currently taught that could be expected in future medical models of care by utilizing the Interpersonal and Communication Skills 8 (ICS8) subcompetency: Act in a consultative role, including participation in the provision of clinical care remotely via telemedicine. Students received no formal training on how to complete a telemedicine visit, but they were given the opportunity to electively review the ICS8 competency grading form (Appendix E) if they wished to be most prepared for interacting with the technology.

Our team created a remote version to allow for instructing and/or assessing learners at a distance from large medical campuses in a standardized way. We assessed learner skill in using the telemedicine technology as a mechanism to augment medical care, as well as learner communication and clinical reasoning skills. We created the Insomnia–Rural TeleOSCE case scenario to depict a patient with depression as an underlying cause for the patient's insomnia (Appendix A). Clinical faculty experienced in diagnosing and treating depression developed the medical knowledge checklist and case scenario. Of note, this was one of many cases in our TeleOSCE curriculum, and cases were rotated regularly to offer variety to learners and maintain the integrity of each student's experience (i.e., avoid students knowing the answers ahead of time).

Institutional review board (IRB) approval was obtained to study the curriculum. An American Board of Internal Medicine Foundation Putting Stewardship Into Medical Education and Training grant partially funded faculty to develop and disseminate TeleOSCE cases incorporating the Choosing Wisely concept[9] of reducing unnecessary medical cost.

We instructed students to read a prompt outside of the room (Appendix B) and then complete the case in 11-13 minutes (depending on scheduling and number of learners). The learners would enter the room to find an SP already connected via live video streaming on a monitor in the room (Appendix C), as well as a faculty observer who did not interact during the case. The faculty member observed the interaction utilizing a standard checklist.

We developed the flow of the case to ensure standardized tasks:

- The student introduces himself or herself and ensures that he/she and the patient can see and hear each other.
- The patient adjusts the monitor to allow better visualization, ideally prompted by the learner.
- The learner asks about the chief concern of insomnia, gathering the patient's pertinent history and sleep hygiene habits, and reviews Patient Health Questionnaire–9 (PHQ-9), a depression screening tool, which the patient has completed electronically prior to the encounter.
- The student is expected to recognize that the patient has moderate depression as a likely cause for the insomnia, to ensure that there is no suicidal ideation, and to recommend counseling and/or medications.
- The SP becomes more irritable because he/she has not been sleeping and reiterates wanting a prescription sleep aid because he/she lives in a rural area without easy access to counselors.
- The student acknowledges the patient's concern, as well as limitations to accessing health care due to the rural setting, and gains buy-in from the patient that helping the depression will help the sleep.

Copyright © 2019 Cantone et al. This is an open-access publication distributed under the terms of the Creative Commons Attribution license.

The case scenario assessed the learner's ability to gather a thorough history in order to delineate likely causes of insomnia, to interpret a PHQ-9 correctly, to interpret the patient's mood appropriately and respond sensitively to it, to formulate an assessment including depression as the likely cause of insomnia, and to negotiate a plan including safe follow-up and counseling (with or without medications). We developed the scoring to include the discussion of depression with the patient as well as determining an appropriate medical plan. We also assessed whether the student acknowledged the patient's frustrations, discussed treatment options that incorporated the patient's rural setting, and negotiated with the patient both an immediate plan and a follow-up plan. There was a checklist item for acknowledging the specific limitations the patient had regarding coming to in-person care in the office due to his or her geographic location. Students were immediately given feedback—from both the SP and the faculty observer in the room. Remotely located learners received the feedback via videoconferencing.

Utilizing the checklist scores, our clerkship team completed the competency assessment (the ICS8) for all students to have added to their competency portfolio. Students who achieved three or more checklist items in the Use of Technology section received an "Entrustable" evaluation; those achieving two items received "Approaching," and those achieving fewer than two received "Pre-Entrustable."

For on-campus learners, we implemented this case as part of a multiple-case OSCE morning that occurred over the course of 4 hours and ended with a debrief led by the course director. Four different TeleOSCE cases, one of which was this insomnia case, were rotated to offer variety. The same software and SPs were used whether the student was on campus or remote. For students in remote locations, this case was designed as one of two in the TeleOSCE format. We instructed learners to review their checklists to understand their performance both clinically and regarding their interaction with the software. We had students complete this OSCE early in their clinical rotation (by the second of 4 weeks) so that they could implement the feedback in the latter half of their rotation.

We developed the checklists to measure three skill areas: communication skills, medical knowledge and clinical reasoning skills, and telemedicine skills (Appendix D). The telemedicine checklist items aligned with national standards created by the American Telemedicine Association, as well as with patient-centered standards on use of technology derived by faculty experienced with those skills. The Insomnia–Rural TeleOSCE also assessed whether a learner acknowledged the limited resources of rural communities in the proposed plan.

We made the evaluation checklist a yes-or-no construct, and we provided required SP and faculty training from the course director prior to the scenario, including at least one observed session, to assess appropriate feedback. There was high variability in the final scores for the overall performance (rated 1-10), but this was not specifically studied as no final grades were given to the students—only verbal or written feedback.

Because the assessment was formative, learners received additional educational resources depending on the areas of improvement identified during the OSCE, including the use of telemedicine or clinical and communication skills. TeleOSCE cases could be used as summative assessments if telemedicine competencies are taught prior to the assessment.

Training Needs

The SPs received formal training prior to attending the session. They had been trained as SPs by our simulation center team prior to being recruited to this case. We created an in-depth instruction manual detailing the case, the learner expectations, and how to address different questions or responses from the learner (Appendix E). We instructed the SPs to focus their feedback on the learner's communication skills and not on checklist items.

The faculty observers received the case (Appendices B, C, and E) and the checklist (Appendix D) ahead of time. They were not required to be experienced with the telemedicine or videoconferencing technology. We trained the family medicine faculty to emphasize the observation of the student-patient interaction using technology. Faculty observation included assessment of history taking, clinical decision making, and shared decision making, and emphasized positive patient interactions despite intentional technological difficulties throughout the scenario.

Personnel and Equipment

- One family medicine faculty, in person or connecting remotely.
- One SP connecting remotely, simulating his or her home.
- One medical student, in person or connecting remotely.
- One staff member monitoring the connection and troubleshooting information technology issues as needed.
- Clinical data (a completed PHQ-9) uploaded onto the software.
- Checklist and case materials for observing faculty.

Copyright © 2019 Cantone et al. This is an open-access publication distributed under the terms of the Creative Commons Attribution license.

Student Assessment

We assessed all students with a standardized checklist on a 1-10 scale, with additional verbal feedback given to the student by both the SP and the faculty at the end of the interaction. The 10-point scale was chosen to best differentiate student performance across a wide range, compared to smaller scales, as we had students in their second, third, or fourth year completing this rotation. If choosing to use this resource as a grade, one might consider a tighter scale to better interpret scores between different faculty observers. We conducted a more in-depth debrief after all students had completed the sessions. Multiple cases were used throughout the years of this study, and the data were not segregated by case to achieve higher numbers in learners engaged in the TeleOSCE experience as a whole.

The TeleOSCE assessed the following competencies, related to the AAMC undergraduate medical education competencies[10] as adopted with changes by our institution:

- Interpersonal Communications Skills:
  - Act in a consultative role, including participation in the provision of clinical care remotely via telemedicine.
  - Communicate effectively with patients across a broad range of socioeconomic and cultural backgrounds.
  - Counsel and empower patients to participate in their care, enable shared decision making.
  - Demonstrate insight into emotions that allow one to develop rapport and manage interpersonal interactions.
- Patient Care and Procedures:
  - Interpret and critically evaluate historical information and data required for diagnosis.
  - Develop and revise, as indicated, patient management plans.
- Medical Knowledge:
  - Apply principles of social-behavioral sciences to assess the impact of care seeking, as well as barriers to and attitudes toward care.
- Professionalism and Personal and Professional Development:
  - Demonstrate responsiveness to a diverse population including socioeconomic status.
  - Demonstrate respect for patient autonomy.
- Systems-Based Practice and Interprofessional Collaboration:
  - Incorporate considerations of resource allocation, cost awareness, and risk-benefit analysis in patient care.

TeleOSCE Evaluation

Students during our study period initially gave informal feedback at the end of the experience during their debrief of the cases. A formalized end-of-rotation evaluation was then formed, which asked students to comment on the “Quality of OSCE Faculty Feedback,” “OSCE Comments/Suggestions,” and “What impact, if any, did the OSCE experience have on your understanding of how to balance value with cost of care (for example: when and why to order imaging, order labs, or order consultations)?” The bottom of the evaluation form asked students to rate the overall experience from 1 to 10 via discrete radio buttons. Student comments were aggregated by theme; this process included two separate iterations of feedback forms due to our institution changing reporting interfaces during the study period.

When our institution again changed the feedback platform, we altered our question to better target addressing the utility of our TeleOSCE and asked students to comment on the “Quality of the lecture/seminar,” keeping the 1-10 radio button scale for consistency.

After gathering and reviewing all of these comments, we pulled out those that specifically addressed telemedicine or the insomnia case and created common themes that were represented in them. Longer comments may have included more than one theme and were separated into two or more comments.

Results

Throughout the course of this study, nearly 500 students in 4 years completed a TeleOSCE experience. We had recorded feedback from 287 students via our end-of-rotation surveys (either the remainder did not complete the survey or their results were unavailable due to changes in data ownership and software platforms). This feedback included mostly third-year students, with some second- and fourth-year students, but demographic data were not collected to protect student anonymity. Ninety-five students were asked specifically about the quality of the lecture/seminar (53 relevant student comments), and 192 were asked the three consecutive questions as listed above (149 relevant comments). An estimated half of these students completed the TeleOSCE insomnia case versus our other TeleOSCE cases. Comments were then separated if they had longer responses that incorporated different themes, achieving a total of 231 discrete comments across all groups. We tallied the comments by hand into common themes, with examples, as listed in the Table.

Copyright © 2019 Cantone et al. This is an open-access publication distributed under the terms of the Creative Commons Attribution license.

Table. OSCE Feedback Themes From End-of-Rotation Survey

Theme	Frequency (out of 231 Comments)	Example Comments
Great experience OR helpful OR fun	61	• "It was a good experience working with telemedicine."<br>• "I liked the telemedicine session as that was a good opportunity for us to get our feet wet in a no-stakes environment!"<br>• "I enjoyed the telemedicine station where we had to figure it out on the fly."<br>• "Helpful tips in using technology and telemedicine concerns."<br>• "I enjoyed being able to practice telemedicine, I think it would be a valuable experience for everyone to try."
Wanted teaching about telemedicine before OSCE	35	• "More instruction or training on how best to practice telemedicine would be a welcome addition."<br>• "It would be nice to have some sort of orientation to TeleOSCE and the software before being thrown in. I felt it was a stressful experience."
Improved understanding of the difficulty of accessing care in rural areas OR addressed barriers/cost to care	28	• "Telemedicine is a unique opportunity to cross multiple barriers, including cost and distance, to patient care."<br>• "The telemedicine OSCE taught me . . . how much it can cost, both in dollars and time, for patients in rural areas to go to the hospital, clinic, and/or pharmacy."<br>• "Telemedicine seems like a useful healthcare tool that, in the right situations, might help strike a balance between value and cost."<br>• "Telemedicine can be an effective method to bring value to patients who live far from a clinic/area with shortage of physicians for less cost."
Concern there will not be future training for telemedicine OR had no prior experience	25	• "Excellent opportunity to use a technology I had never had a chance to use before."<br>• "Very useful in seeing how telemedicine is practiced! It was nice to have this experience before actually trying telemedicine in the real world."
Important and/or difficult, challenging but educational	23	• "It was a difficult medium to do a patient exam but I can see the usefulness in reaching remote patients with no other access to care."<br>• "Challenging as well as informative for using the technology."
Appreciation of differences between in-person and remote visits	16	• "I do appreciate that remote discussions with providers can give enough info if the patient is a good historian, but I miss the personal connection. Building a trusting relationship with a patient may be harder."<br>• "It taught me how to assess triaging in a virtual setting. It allows me to be able to talk with patients and focus on building a rapport in a novel medium."<br>• "This experience cautioned me to be more aware of my internal bias toward in-person visits vs telemedicine calls."
Want fewer distractions (clinical or technical), difficult to provide care	15	• "Maybe a slightly simpler case for students . . . so that they can focus more on the technology."<br>• "I see that telemedicine can be a way to overcome a barrier to health care, but I found it difficult to provide quality care."<br>• "I think that the telemedicine OSCE was really tricky. I definitely appreciate the use of this technology for accessing more rural communities or people who could not otherwise access care. However, I felt like it was a slower and more cumbersome process for the provider and I definitely felt uncomfortable not being able to examine the patient."
Worry about what they would be judged on or how they were graded	8	• "Was unsure whether we were allowed to click on computer. Would have appreciated one line on the prompt sheet that says that the computer is 'in play.'"<br>• "Tell students that we are being evaluated on HOW we use the technology—we thought it was more about how we took the history, etc., but necessarily knowing that we were being critiqued on how we utilized telemedicine."
Had technical difficulties but seemed realistic OR learned the limitations	5	• "It helped me understand that tele-encounters are done out of necessity and unfortunately have several drawbacks."<br>• "Again, it was useful to practice using telemedicine in a low stakes environment. It was pretty awkward so having that exposure was really helpful."
More time is needed	5	• "It wasn't enough time to practice telemedicine skills, nor was there enough context for it to be a very effective educational experience."<br>• "I think more time needs to be allotted due to unforeseen technological issues."<br>• "A little more time to work with the system/read the case before the patient was online would have been appreciated."
Skeptical of utility	5	• "I am not yet entirely convinced about telemedicine and getting the same value out of this visit versus the telemedicine visit. It is more difficult to develop a strong physician-patient relationship, so I think I need a bit more experience to understand the value and trade-offs for telemedicine."
Telemedicine as triage	5	• "Appreciate that it can be used as a triage function and save families difficult or time consuming/gas consuming trips."

Copyright © 2019 Cantone et al. This is an open-access publication distributed under the terms of the Creative Commons Attribution license.

Students rated the overall experience on a scale of 1-10. The average student response was 7.59 out of 10. Some students in the group were asked to rate the quality of faculty feedback, comments, and suggestions, as well as the impact of the value/cost of care. Their ratings averaged 7.34, which was lower than ratings by students asked simply about the quality of the lecture/seminar, which averaged 8.10.

During the debrief, students commented that they had learned how to better connect with patients remotely, including how to make eye contact via a webcam. They also commented on the subtleties of assessing mood and affect over a computer screen and how they needed to think about these differently from an in-person visit. Learners felt that this case was a good experience for a reasonable telemedicine visit that did not require a full physical examination.

However, students with experience with telemedicine commented in the debrief that the case may not have been an accurate depiction of the telemedicine software used elsewhere in our community. Several students showed skepticism about the utility of this skill, and many requested more specific training before the scenario or fewer distractions by either the technology or medical questions during the case.

Faculty commented to the course director (verbally and not formally collected) that they enjoyed giving students immediate feedback. Faculty who worked away from the main campus reported appreciation for the ability to interact with medical students from their home or office.

Discussion

The TeleOSCE fulfills several needs: It exposes learners to the concepts of telehealth, assesses clinical and diagnostic reasoning skills of remote/rural learners, engages remote/rural faculty wanting to teach, assesses learners on communication skills and medical knowledge, and addresses the need for improved and more frequent telemedicine training.

The TeleOSCE has been mostly well received by students. Faculty can observe students from remote locations, home or clinic, allowing expansion of our teaching faculty free of geographic limitations. This would work well for dispersed campuses across the US and abroad. Rural practitioners have continued to sign up for this experience to remain engaged in undergraduate medical education. This tool could be used for preclinical students, national assessments such as licensing examinations, and resident evaluations, or by practicing health care providers needing feedback on and assessment of their patient care skills.

Student feedback showed us several ways to improve this experience. Notably, students wanted more exposure before the scenario itself, more specific instructions on the evaluative tools, more time to complete the scenario, and fewer technical difficulties and medical distractions. We believe that in real life, providers do not always know why the patient is seeking care and remain under tight time pressures to improve patient access. However, we may liberalize the time allowed for students to more completely grasp the technology during the scenario. Fortunately, many students commented on the experience being helpful or positive, as well as useful for understanding limitations in accessing care due to cost or location, and mentioned that they better appreciated the difference between a remote and an in-person visit. Others remained skeptical of the utility or treated it mostly as a triage situation. Most worried that they had not yet received training in telemedicine and might not receive further training in the future.

Limitations exist related to cost and implementation. We used paid SPs, which may be cost prohibitive in certain situations. Faculty or pairs of learners may suffice to offset this need but could decrease the rigor of the TeleOSCE. Some learners and faculty may not be comfortable with the technology, so support staff should be well trained and present to help with the flow and any real technological limitations (as opposed to what the learners are being assessed on). As we are reaching rural locations, connectivity issues with cell phones and the internet can significantly affect the encounter. We have mitigated this at times by use of a landline telephone and wired internet connection. Learners and SPs often accidentally speak over each other due to delayed audio and video feeds, but we believe that this reflects what can occur in real practice and that troubleshooting techniques are important to learn and practice. Multiple videoconferencing options now exist and can be trialed in different locations, although there needs to be a way to ensure that the clinical data are available for the learner.

Additionally, there were limitations with our data collection. We are missing data on several students due to not collecting a survey at certain points in time, lack of completion by the students, and migration of data from different software that became unavailable when reviewing the data for this study. We also did not collect demographic data to maintain student confidentiality as per our IRB approval. As the curriculum was designed to provide feedback to students over a variety of different TeleOSCE cases, we did not collect the numerical performance of the 1-10 scale on the bottom of the checklist. We also did not tally the overall performance on the checklist, as the focus was on improving exposure of students to telemedicine.

Copyright © 2019 Cantone et al. This is an open-access publication distributed under the terms of the Creative Commons Attribution license.

We are concerned about how generalizable our findings would be due to our nontraditional curricular structure with second-, third-, and fourth-year students with a wide range of prior experiences taking part in this case. Our qualitative comments came from the debrief and from end-of-rotation evaluations, so we cannot completely sort out if the end-of-rotation evaluations specifically came from the experience itself or the debrief that occurred afterward. Finally, as we initially sought to improve the telemedicine experience, we did not track which case students had completed when we collected their comments. In the future, it would be beneficial to track student performance and comments to better understand differences between the cases, levels of learner, and influence of the debrief session versus the experience itself.

As telemedicine continues to grow, there will be an increasing need to train learners in this type of medical care. Our students and faculty have reported understanding the use and limitations of telemedicine interactions as a valuable outcome of this TeleOSCE. Videoconferencing software options continue to develop and may offer reliable, cost-conscious platforms for use with this type of education for resource-limited institutions or programs. Since our team's general TeleOSCE experience was created, it has resulted in multiple publications examining ways to standardize curricula for remote and rural learners[11] and has shown a positive impact on student education.[1] These publications address the quantitative data collected, whereas the qualitative feedback has led to the development of additional cases. We continue to explore expanding the TeleOSCE casework with other institutions and types of learners to make it a more available assessment method for dispersed educational models.

Appendices

A. Standardized Patient Case.docx

B. Student Scenario.docx

C. Room Setup.pdf

D. Checklist.docx

E. ICS8 Competency Form.docx

All appendices are peer reviewed as integral parts of the Original Publication.

Rebecca E. Cantone, MD: Assistant Professor, Department of Family Medicine, Oregon Health & Science University; Director of Family Medicine Core Clinical Experience (Clerkship), Oregon Health & Science University; Director of Family Medicine Student Education, Oregon Health & Science University

Ryan Palmer, EdD, MFA: Associate Dean of Medical Education, Northeast Ohio Medical University

Lisa Grill Dodson, MD: Campus Dean, Medical College of Wisconsin–Central Wisconsin

Frances E. Biagioli, MD: Professor, Department of Family Medicine, Oregon Health & Science University; Vice Chair of Education, Department of Family Medicine, Oregon Health & Science University

Disclosures
None to report.

Funding/Support
None to report.

Ethical Approval
The Oregon Health & Science University Institutional Review Board approved this study.

References

1. Lacktman NM, Vernaglia LW. 2014 Telemedicine Survey executive summary. Foley & Lardner website. https://www.foley.com/en/insights/publications/2014/11/2014-telemedicine-survey-executive-summary. Published November 11, 2014.
2. Nesbitt TS, Dharmar M, Katz-Bell J, Hartvigsen G, Marcin JP. Telehealth at UC Davis—a 20-year experience. *Telemed J E Health*. 2013;19(5):357-362. https://doi.org/10.1089/tmj.2012.0284
3. Beal MD, Kinnear J, Anderson CR, Martin TD, Wamboldt R, Hooper L. The effectiveness of medical simulation in teaching medical students critical care medicine: a systematic review and meta-analysis. *Simul Healthc*. 2017;12(2):104-116. https://doi.org/10.1097/SIH.0000000000000189
4. Shortridge A, Steinheider B, Ciro C, Randall K, Costner-Lark A, Loving G. Simulating interprofessional geriatric patient care using telehealth: a team-based learning activity. *MedEdPORTAL*. 2016;12:10415. https://doi.org/10.15766/mep_2374-8265.10415
5. Biery N, Bond W, Smith AB, LeClair M, Foster E. Using telemedicine technology to assess physician outpatient teaching. *Fam Med*. 2015;47(10):807-810.
6. Palmer RT, Rdesinski RE, Galper A, et al. Assessing the impact of a telemedicine simulation on clinical learners. *J Fam Med Community Health*. 2017;4(5):1120.
7. Palmer R, Dodson L, Biagioli F. Telemedicine OSCE ("TeleOSCE"): rural diabetes. Society of Teachers of Family Medicine's National

Copyright © 2019 Cantone et al. This is an open-access publication distributed under the terms of the Creative Commons Attribution license.

Clerkship Curriculum website. https://www.stfm.org/teachingresources/curriculum/nationalclerkshipcurriculum/acutechronicpreventivecarecurricula/#7332. Accessed November 20, 2018.

8. Obeso V, Brown D, Aiyer M, et al; for Core EPAs for Entering Residency Pilot Program. *Core Entrustable Professional Activities for Entering Residency: Toolkits for the 13 Core EPAs*. Washington, DC: Association of American Medical Colleges; 2017.

9. Choosing Wisely website. http://www.choosingwisely.org. Accessed November 18, 2018.

10. *Core Entrustable Professional Activities for Entering Residency: Curriculum Developers' Guide*. Washington, DC: Association of American Medical Colleges; 2014.

11. Palmer RT, Biagioli FE, Mujcic J, Schneider BN, Spires L, Dodson LG. The feasibility and acceptability of administering a telemedicine objective structured clinical exam as a solution for providing equivalent education to remote and rural learners. *Rural Remote Health*. 2015;15(4):3399.

Received: January 31, 2019
Accepted: September 7, 2019
Published: December 27, 2019

Copyright © 2019 Cantone et al. This is an open-access publication distributed under the terms of the Creative Commons Attribution license.
