# Supplementary material for: Insomnia Telemedicine OSCE (TeleOSCE): A Simulated Standardized Patient Video-Visit Case for Clerkship Students
Source: MedEdPORTAL. 2019 Dec 27;15:10867. doi: 10.15766/mep_2374-8265.10867 (PMC7012306; doi:10.15766/mep_2374-8265.10867)
Supplement: Supplementary file 1 — A. Standardized Patient Case.docx B. Student Scenario.docx C. Room Setup.pdf D. Checklist.docx E. ICS8 Competency Form.docx [file mep-15-10867-s001.zip › A. Standardized Patient Case.docx]

Appendix A: *MedEdPORTAL* Standardized Patient Case Development Tool: Telemedicine Insomnia

Date: 6/18/19

Primary Case Author: Ryan Palmer, EdD, MFA

Secondary Case Author: Brian Frank, MD, Lisa Dodson, MD, Frances Biagioli, MD, Rebecca Cantone, MD

Standardized Patient Educator: Ryan Palmer, EdD, MFA, Rebecca Cantone, MD

Name of Case: Insomnia-Rural

Name of educational and or assessment activity: Telemedicine OSCE (TeleOSCE)

Patient Name: Lou Lewis

Chief Complaint: Insomnia

Most likely Diagnosis and Differential with rationale from history and/or physical exam:

Major Depressive Disorder, Moderate: Patient has a PHQ-9 completed screening positive for depression, a low energy voice, a depressed affect, and difficulty sleeping since the death of their partner.

Challenge question: What do you offer this patient to treat the insomnia?

Domains: Check all that apply

X Professionalism

X Communication and Interpersonal skills

X Medical History

- Physical exam

X Shared Decision Making

X Patient Education

X Clinical Reasoning

- Documentation
- Handoff
- Presentation

X Other: Telemedicine Skills

Type and level of learner: Medical Students on the Family Medicine required clinical-year rotation

Case Objectives: please list specific objectives for each of the domains you have checked above:

1. Incorporate geographical limitations of care into patient care plans
2. Demonstrate the provision of clinical care remotely via telemedicine.
3. Use clinical decision support tools to improve the care of patients.
4. Recognize the use of telemedicine video technology to clinically assess a patient with insomnia
5. Identify the care needed for a patient with insomnia due to depression

| SETTING: | The patient is at home. The learner is utilizing a telemedicine visit from their home or office |
| --- | --- |
| PATIENT PROFILE: Lou Lewis is a 68yo contacting the family physician via remote telemedicine from a home computer for difficulty sleeping for the past few weeks. | |
| Age range | 50-75 |
| Religious/spiritual background | All may be used |
| Sex (e.g., male, female, intersex, transwoman, transman) | All may be used |
| Sexual Orientation (e.g., heterosexual, lesbian, gay, bisexual, pansexual, queer, asexual) | All may be used |
| Gender expression (e.g., man, woman, gender queer) | All may be used |
| Race/ethnicity: | All may be used |
| Physical description (e.g., BMI, height range) | All may be used |
| Physical limitations | NONE |
| Patient appearance (e.g., disheveled, hospital gown, business casual, casual) | Tired, in sleepwear, poorly groomed |
| Moulage + location (e.g., none, bruises, scars, body piercing, tattoos) | All may be used |
| Affect (e.g., pleasant, cooperative) | Blunted, fatigued |
| Family group (e.g., who is family, who they live with) | Lives alone, partner died a few months ago |
| Education | Associates Degree |
| Level of health literacy | Fair – does not understand medical jargon |
| Employment, if any - present and past, noting any current stresses | Retired from Office Manager for a trucking company |
| Home/homeless - type of dwelling, number of stories, owned or rented | Housed – can be house or apartment of any style |
| Financial situation- any current stresses | This patient lives in a remote rural area and has limitations to transportation – they cannot easily travel in to clinic without a lot of coordination of schedules with family |
| Insurance Status (e.g., un/under/insured, public/private, HMO/PPO) | Medicare |
| Habits (i.e., diet, exercise, caffeine, smoking, alcohol, drugs) | No smoking, 3 glasses of wine maximum per week, no tobacco or alcohol |
| Activities (i.e., hobbies, sports, clubs, friends) | Visiting son in Boise, sometimes volunteers at nearby school |
| Typical day - what is the usual daily routine | Mostly at home, no activities |

| CASE INFORMATION | |
| --- | --- |
| Chief Concern: | “I’ve been having trouble sleeping and now I feel tired all day. I’m hoping you can help me figure out what’s going on.” |
| Additional Concerns: Other, if any, concerns the patient has today (i.e., symptoms, requests, expectations, etc.) that will become part of set agenda. | I would like something for sleep. |
|  | |
| THE PATIENT STORY: | Last month I started having trouble sleeping. Usually I go to bed around 9 PM and fall asleep shortly thereafter. About a month ago, I began to have trouble falling asleep. I often lie awake well past 10 PM and sometimes later. When I do finally fall asleep, I wake up frequently tossing and turning. I’ve tried everything to make falling to sleep easier. I don’t watch TV in bed, don’t drink alcohol immediately before you go to bed and I have tried reading as a way to help fall asleep. Nothing has worked. I now feel tired most of the day as a result and have finally decided to talk to someone about it.  Besides the trouble sleeping, I have no health issues. |
| HISTORY OF PRESENT ILLNESS: | |
|  | |
| Onset (when; gradual or sudden) | Last month, gradually noticed |
| Setting (what was going on or where was patient when symptoms first noticed?) | Usual bedtime 9pm but now lie awake well past 10 or later |
| Duration (how long) | Every night for the month |
| Time relationships (frequency, constant or intermittent) | Every night |
| Location | n/a |
| Radiation | n/a |
| Quality | n/a |
| Amount | n/a |
| Aggravated by what | Lying in bed |
| Relieved by what | Nothing including alcohol, TV or reading |
| Associated with what | No TV in bed, no alcohol before bed, reading doesn’t work |
| Attitude (what does the patient think is the problem, and how does he/she feel about it) | Patient is not sure what is causing this. If the learner suggests depression is possibly the cause “I never thought it could be from depression, why do you think that?“ |
| Overall course | I live alone and have been widowed for just over a year now. I retired as an office manager for a trucking company and we moved to a small town for retirement. My partner died unexpectedly last year from an infection. We were very close and had planned on living many more years together. I’ve gone through many stages of grieving and don’t really feel like I’ll ever be “over it.” I have one son who lives in Boise with his wife and they have a 5-year-old daughter but do not see them as often as I would like. We Skype weekly and see each other about twice a year and I don’t have a strong support network in town. Sometimes I volunteer at the local school but have done so less since my partner died. Sometimes I feel very lonely and think about selling the house and moving closer to Boise, but the housing market is bad and I don’t want to burden my son. Sometimes I feel stuck.  . |
| REVIEW OF SYSTEMS: Significant positives and negatives | |
| Psych: | No Suicidal or Homicidal Ideation, mood is low |
|  | |
| Past medical history |  |
| Medication allergies (Name and reaction) | None |
| Environmental allergies (Name and reaction) | None |
| Illnesses | None |
| Vaccinations | Up-to-date |
| Surgeries | None |
| Accidents/ injuries/ trauma | None |
| Hospitalization | None |
|  | |
| Inclusive sexual and reproductive history | |
| Sexual practices  Sexual partners  Protection: Use of safer sex practices  Use of birth control if appropriate  Risk of intimate partner violence | Was sexually active with prior partner only, no new partners since spouse passed away. Can be any gender or sexuality, no risk of pregnancy (either postmenopausal or partner cannot get pregnant) |
| Ob/GYN HISTORY | If gender of actor is female:  Age of onset of menses: “a long time ago”  Age of menopause: 55  Number of pregnancies: 1  Number of live births: 1  Number of miscarriages: 0  Number of abortions: 0 |
| Medications | No prescriptions or OTC Medications |
| Immunizations | X Tetanus  X Flu  X Hepatitis  X Pneumovax   - HPV - Other |
| Tobacco products:   - Cigarettes - Cigar - Pipe - Chew - E-cigarettes | X Never   - Past- year started/year quit - Current   - Quantity   - # of years |
| Alcohol   - Beer   X Wine   - Liquor - Other | - Never - Past- year started/year quit   X Current   - - Quantity: 3 glasses of wine per week   - # of years: less than a year. |
| Drugs   - Weed - Cocaine - Heroin - Meth - Other - IV - Inhalants - Other | X Never   - Past- year started/year quit - Current   - Quantity - # of years |
| Diet (describe) | No restrictions |
| Exercise (describe) | Minimal |
| List any other important social history or information important to this case | Refer to “Overall Course” above |
| Family history |  |
| Mother, Father, Siblings, Grandparents, and other significant findings. | Parents lived until 90s and died of “natural causes” |
|  |  |
| Physical Exam-  No vitals collected as patient does not have supplies at home.  Exam limited to general appearance and observation | |
| PHYSICAL EXAM FINDINGS |  |
| 1. Written in layman’s terms | Quiet, irritable, tired |
| 1. General appearance- affect, appearance, position of patient at opening (i.e. sitting, laying down, holding abdomen etc.) | Alert, low energy in voice, wearing sleepwear, sitting at computer |
| 1. Vital signs | NONE |
| 1. Specific findings and affect | Sad affect, irritable tone, normal speech and judgement, good eye contact |
| 1. Response to certain physical movements | Not applicable |
|  |  |
| DIAGNOSIS AND DIFFERENTIAL |  |
| Diagnosis with support from positive and negative history and PE findings | Major Depressive Disorder – patient reports death of partner, “the bed just feels a bit bigger now since they died.” PHQ-9 results interpreted along with affect examination should lead to a diagnosis of depression. |
| Differential with support from positive and negative history and PE findings | Alcohol Use Disorder – unlikely given low report of wine consumption  Thyroid Disease – unlikely given lack of family history and medical care previously up to date |
|  |  |
| MANAGEMENT OR DIAGNOSITIC PLAN | Recommend counseling near home (due to transportation issues getting to clinic) and/or offering a connection with the clinic’s behavioral health team resources, and/or recommend a first line antidepressant such as a selective serotonin reuptake inhibitor (SSRI) |
|  |  |
| PROFESSIONALISM ISSUES OR CHALLENGES: | Address the patient’s concerns of not sleeping by diagnosing depression and communicating that in a compassionate matter that does not convey stigma |

Further instructions for SP (Text)

Family Medicine Clerkship OSCE

Telemedicine- Sleep Problems

Patient Scenario

You are Lou Lewis, a 68-year-old who is contacting your family physician via a remote telemedicine hookup from your home computer. The nature of your consult is you have been having trouble sleeping for the past few weeks. You’re increasingly tired all day and so you emailed your physician the night before and he/she set up this telemedicine consult with you.

You live in the small town of Juntura, OR. Your family physician, Dr. Smith, is 1 hour away in Burns, OR. It is due to this remote location that your physician set you up with a telemedicine option in your home, which is accessible by logging into a secure web portal on your computer. You have satellite Internet connectivity that is excellent, allowing you to participate in the telemedicine program. You’ve used the technology to communicate with your family physician before and feel comfortable using the interface.

Last month you started having trouble sleeping. Usually you go to bed around 9 PM and fall asleep shortly thereafter. About a month ago you began to have trouble falling asleep. You often lie awake well past 10 PM and sometimes later. When you do finally fall asleep, you wake up frequently tossing and turning. You now feel tired most of the day as a result and have finally decided to talk to someone about it. You hope the doctor can help diagnose why you aren’t sleeping well and give you something that can help.

The student in this scenario will be asked to take a pertinent history from you and develop an action plan. The student is filling in for your regular physician, Dr. Smith. You like your new doctor and appreciate the use of technology to prevent you from having to always drive in to clinic. The student has never met you before.

Your opening statement will be: “I’ve been having trouble sleeping and now I feel tired all day. I’m hoping you can help me figure out what’s going on.”

History of current illness

Last month you started having trouble sleeping. Usually you go to bed around 9 PM and fall asleep shortly thereafter. About a month ago you began to have trouble falling asleep. You often lie awake well past 10 PM and sometimes later. When you do finally fall asleep, you wake up frequently tossing and turning. You’ve tried everything to make falling to sleep easier. You don’t watch TV in bed, you don’t drink alcohol immediately before you go to bed and you’ve tried reading as a way to help you fall asleep. Nothing has worked. You now feel tired most of the day as a result and have finally decided to talk to someone about it. Besides the trouble sleeping, you have no health issues.

Social history

You live alone and have been widowed for just over a year now. Your late partner and you moved to Juntura 5 years ago after they retired from a job as a schoolteacher and you retired as an office manager for a trucking company. You moved to Juntura from LaGrande, OR because you both wanted to live in a small town for your retirement. Your partner died unexpectedly last year from a respiratory infection. The two of you were very close and had planned on living many more years together. You have gone through many stages of grieving and, though you have accepted they are gone now, you do not really feel like you will ever be “over it.” You have one son who lives in Boise with his wife and they have a 5-year-old daughter. You are close with your son and his family but do not see them as often as you would like. You Skype weekly and see each other about twice a year. As you moved to Juntura and the town is quite small, you really don’t have a strong support network in town. Sometimes you volunteer at the local school but have done so less since your partner died. Sometimes you feel very lonely and think about selling the house and moving closer to Boise, but the housing market is bad and you don’t want to burden your son. Sometimes you feel stuck.

Before your partner passed away, you only drank occasionally. Since they passed, you drink 3 glasses of wine a week, though never more. You have never smoked cigarettes or used any recreational drugs. You do not take any medicines regularly.

Family history

Your mother and father both died of natural causes in their early 90s. Your father and your mother had no health conditions. You have no siblings. There is no other history of illness in your family.

Your affect and behavior:

- - - You do not think your trouble sleeping is connected to being depressed. If the student asks if you feel depressed say, “I don’t know. I just thought I was having trouble sleeping.”
    - If the student suggests you are depressed, you will initially disagree and ask him/her how they know. You will only accept that you could be depressed if the student explains the results of the depression-screening tool that you completed before the visit. If the student does this, you will ask him/her to explain the test and the results.
    - You do not have any thoughts of suicide or self-harm (only reveal this if asked by the student)
    - The student should hear fairly quickly that your spouse died last year as this will cue the student that this may be depression. You can indicate your spouse’s death in how you answer the student’s questions. For example, if a student asks why you think you aren’t sleeping well, you can answer, “I’m not really sure. Maybe because the bed just feels a bit bigger now since my husband is gone.”
    - If the student asks about your drinking you can say that your 3 glasses a week of wine is definitely more that you used to drink before you spouse passed away.
    - Your demeanor is downbeat. You do not smile easily, and your voice lacks energy.
    - You will answer the student’s questions and not hold back information.
    - If the student asks you to come in for a clinic visit you will be hesitant. You are on a fixed income and gas is expensive, so you would rather not make the hour drive to Burns if you don’t have to. If the student is persistent and makes a good case as to why you should come in, you will ultimately agree to do so.
    - At the beginning of the encounter, place the camera so that it is angled at the top of your head (face off camera) so the student can only see top of your head. If the student does not ask you to adjust your camera after the first minute of the encounter, ask the student if he/she can see you ok. If they still don’t ask you to adjust your camera, do it yourself and give them feedback at the end of the encounter that they should help make sure the patient is fully visible when doing a telemedicine encounter.

Screening Tests

Before meeting with the doctor, the medial assistant had you fill out two screening forms on your computer. If the student asks you about them, just say you can’t really remember what you answered but you think the assistant said doctor can get the results somewhere in the telemedicine room (the student will have to download them to view). You received a 15 on the PHQ-9 scale.

Note: The PHQ-9- Depression screening should be uploaded to the case. We recommend uploading a picture of the PHQ-9 which is free to access (Developed by Drs. Robert L. Spitzer, Janet B.W. Williams, Kurt Kroenke and colleagues, with an educational grant from Pfizer Inc. No permission required to reproduce, translate, display or distribute) or utilize your home institution’s electronic medical record PHQ-9 if one exists.
