# Supplementary material for: Insomnia Telemedicine OSCE (TeleOSCE): A Simulated Standardized Patient Video-Visit Case for Clerkship Students
Source: MedEdPORTAL. 2019 Dec 27;15:10867. doi: 10.15766/mep_2374-8265.10867 (PMC7012306; doi:10.15766/mep_2374-8265.10867)
Supplement: Supplementary file 1 — A. Standardized Patient Case.docx B. Student Scenario.docx C. Room Setup.pdf D. Checklist.docx E. ICS8 Competency Form.docx [file mep-15-10867-s001.zip › B. Student Scenario.docx]

**Family Medicine Clerkship OSCE**

**Telemedicine**

You have a total of 12 minutes to read this scenario and to conduct the telemedicine visit

**Student Scenario**

You are doing a telemedicine consult with Lou Lewis, a 68-year-old who is contacting their family physician via a remote telemedicine hookup from their home computer. You are a physician working as a locums provider in Burns, Oregon filling in for Lou’s primary care physician, Dr. Smith, who is unreachable and out of the country on a medical mission in Nepal for several weeks. Dr. Smith has done several telemedicine consults with Lou, who is familiar with the technology and format of video visits. Lou lives in Juntura, Oregon, which is approximately 58 miles away from the clinic you are currently working at in Burns. Last night Lou emailed you with a complaint of having trouble sleeping for the past few weeks.

Technology:

- You will interact with Lou Lewis via a telemedicine system.

- Your Medical Assistant has administered the annual screening test which you can download from the telemedicine system.

- There are resources for interpreting the results of the screenings available in the Telemedicine system.

**Your Tasks**

*1) Take a pertinent history of present illness (see below for chart review).*

*2) View and interpret annual screening test*

*3) Determine the nature of the issue and recommend treatment and follow up*

Clinic visit

Patient Name: Lou Lewis Med. Rec. No.: 3289

This is a 68-year-old with complaint of having trouble sleeping.

**PMH**: None

**Medications**: None; Allergies, none.

**Habits**: Drinks three glasses of wine week. Has never smoked cigarettes or used any recreational drugs. Patient does not take any medicines regularly.

**Family history**: Mother and father both died of natural causes in their early 90s. Father and mother had no health conditions. Has one son, who lives in Boise. No siblings. No other history of illness in the family.

**Social History:** Patient is single (widowed). Medicare patient with comprehensive health insurance.
