# Supplementary material for: Insomnia Telemedicine OSCE (TeleOSCE): A Simulated Standardized Patient Video-Visit Case for Clerkship Students
Source: MedEdPORTAL. 2019 Dec 27;15:10867. doi: 10.15766/mep_2374-8265.10867 (PMC7012306; doi:10.15766/mep_2374-8265.10867)
Supplement: Supplementary file 1 — A. Standardized Patient Case.docx B. Student Scenario.docx C. Room Setup.pdf D. Checklist.docx E. ICS8 Competency Form.docx [file mep-15-10867-s001.zip › C. Room Setup.pdf]

Telemedicine OSCE Room Set Up

Set up a screensharing video conferencing software system of your choice. Ensure the depression screening PHQ-9 tool is uploaded to a file sharing section of the software.

The result of the PHQ-9 should equal a score of 15/27 (moderately severe depression).

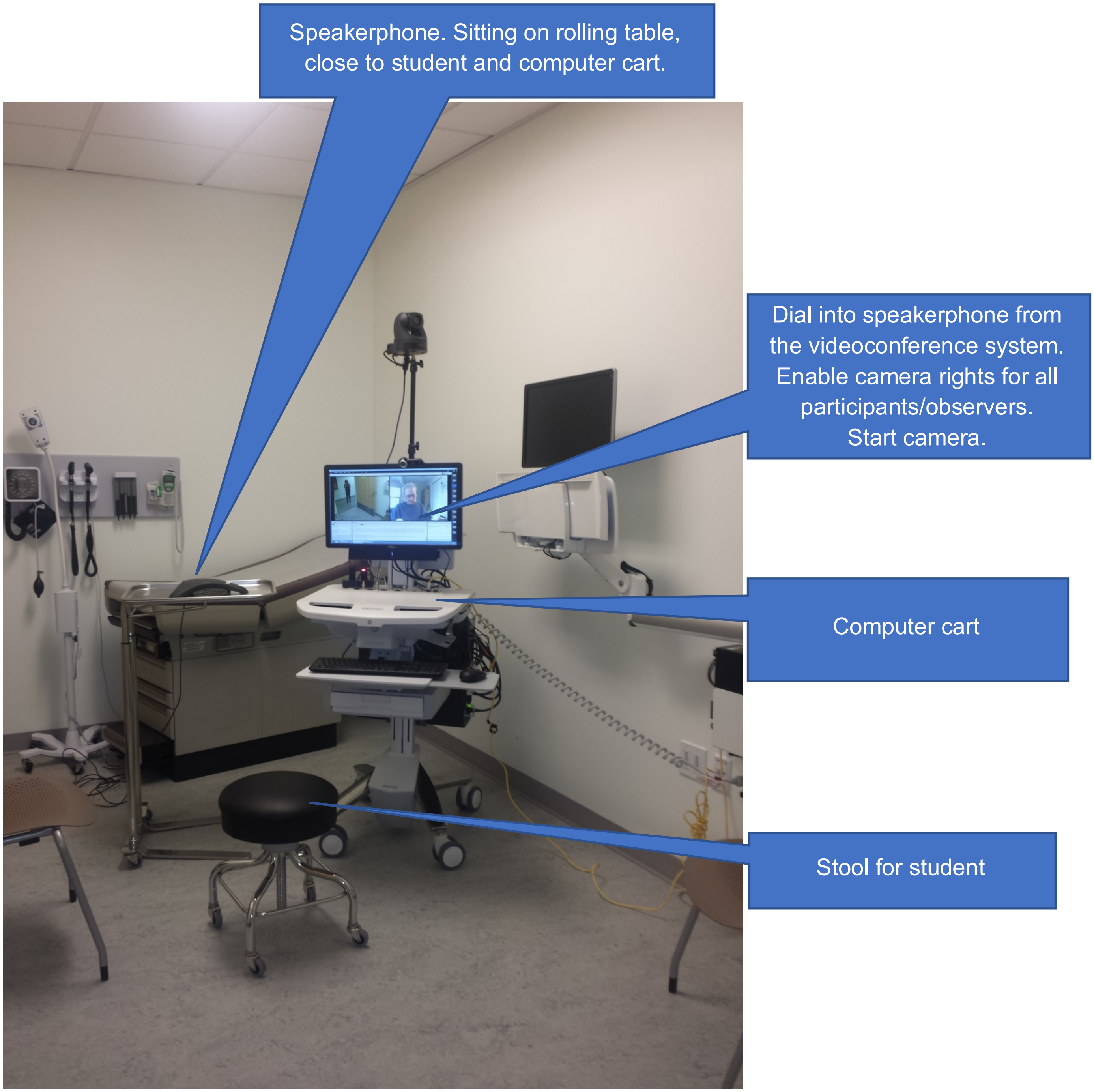

***Image is Author Owned**
