# Supplementary material for: Insomnia Telemedicine OSCE (TeleOSCE): A Simulated Standardized Patient Video-Visit Case for Clerkship Students
Source: MedEdPORTAL. 2019 Dec 27;15:10867. doi: 10.15766/mep_2374-8265.10867 (PMC7012306; doi:10.15766/mep_2374-8265.10867)
Supplement: Supplementary file 1 — A. Standardized Patient Case.docx B. Student Scenario.docx C. Room Setup.pdf D. Checklist.docx E. ICS8 Competency Form.docx [file mep-15-10867-s001.zip › D. Checklist.docx]

**Family Medicine Clerkship OSCE: Telemedicine – Sleep Problems**

**Observer Checklist**

**Student name:** **___________________________________________________**  **Date:** ___________________
*Place a check in front of each task that the student accomplished correctly. Do not place a check for any tasks that were forgotten, missed or done partially or incorrectly. If "and" is used for a task, the student must complete all tasks. If "or" is used for a task, the student must complete or address at least one of the tasks. Students will have 2 minutes to “wrap-up” the interaction, discuss follow-up, and/or provide closure without observer prompting.*

Interpersonal Skills. *The student:*

_____ 1. Introduced self to patient.

_____ 2. Established rapport at the beginning of the encounter.

_____ 3. Allowed the patient to answer questions without interruption or interjection.

_____ 4. Used language the patient could understand.

_____ 5. Expressed empathy (e.g. transportation issues, acknowledged patient frustration).

_____ 6. Verbalized clear instructions for next steps in care following this electronic encounter.

_____ 7. Ask the patient if he had any additional questions or concerns.

_____ 8. Summarize the encounter including having the patient teach back the recommendations.

Clinical knowledge. *The student*:

_____ 9. Identified depression as a possible cause of trouble sleeping.

_____ 10. Student ensures that patient has no thoughts of suicide or self-harm

____ 11. Student screens for a history of mania

_____ 12. Student asks about history of substance and alcohol use

_____ 13. Student determines appropriate treatment plan based on the PHQ-9’s indicated level of depression

_____ 14. Student uses shared decision making with patient to determine treatment plan

_____ 15. Student provides a clear follow up plan (i.e. “we should talk again in xx days/weeks)

Use of Technology: *The student*

_____ 16. Asked the patient if he could see and hear with the technology.

_____ 17. Makes any necessary adjustments and to accommodate for technologic issues. (asks patient to adjust camera)

_____ 18. Accessed the patient’s PHQ-9 in using the telemedicine interface.

_____ 19. Verbalized what he/she was doing when not focused on the patient (e.g., while viewing PHQ-9 results).

_____ 20. Remained patient-centered despite distractions or technology interface challenges (acknowledges any

distractions due to technology and explains what is going on to patient)

Comments for the student:

**Follow-up:**  Would any further learning activities be helpful to this student? Yes/No

Specific skills to address:

**OVERALL EVALUATION (circle below):**

*Students are not graded on these stations; however, to assist with individual feedback, please evaluate their overall performance.*

1 2 3 4 5 6 7 8 9 10

Faculty Observer: _____________________________________________________________________________
