# Supplementary material for: Insomnia Telemedicine OSCE (TeleOSCE): A Simulated Standardized Patient Video-Visit Case for Clerkship Students
Source: MedEdPORTAL. 2019 Dec 27;15:10867. doi: 10.15766/mep_2374-8265.10867 (PMC7012306; doi:10.15766/mep_2374-8265.10867)
Supplement: Supplementary file 1 — A. Standardized Patient Case.docx B. Student Scenario.docx C. Room Setup.pdf D. Checklist.docx E. ICS8 Competency Form.docx [file mep-15-10867-s001.zip › E. ICS8 Competency Form.docx]

**Competency: Interpersonal and Communications Skills #8**

Act in a consultative role, including participation in the provision of clinical care remotely via telemedicine or other technology.


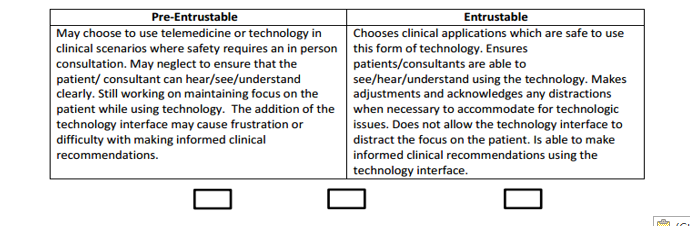


**Total score: ________________**

Measure: Telemedicine OSCE Simulation

Write in the number of checkmarks in the Use of Technology section of the OSCE Checklist.

If students do more than one station, use the highest score attained

Scoring of Entrustment level

Use of Technology Section of the Checklist (Note, some scenarios have 5 some 6 questions):

4-6 Checkmarks = Entrustable

3 checks = Approaching Entrustable

0‐2 check marks: Pre‐ entrustable
